# Eco friendly obtained zirconium oxide crystals for efficient separation of rare earth elements from acidic media

**DOI:** 10.1038/s41598-026-48985-3

**Published:** 2026-05-09

**Authors:** A. El-Tantawy, I. M. Ali

**Affiliations:** Nuclear Fuel Technology Department, Hot Laboratories and Waste Management Center, Egyptian Atomic Energy Authority, P.O. 13759, Cairo, Egypt

**Keywords:** Green synthesis, Adsorption, ZrO_2_, Lanthanides, Kinetic, Isotherm, Chemistry, Environmental sciences, Materials science

## Abstract

This study examined synthesis of zirconium oxide (ZrO_2_) using a straightforward green synthesis method and its application for preferential adsorption of La^3+^, Eu^3+^, and Sm^3+^, demonstrating a low-cost and environmentally friendly approach. Several analytical techniques were employed to characterize (ZrO_2_) specifically, XRD, TGA-DTA, SEM-EDX, and FTIR. Through batch mode experiments, the effectiveness of ZrO_2_ to adsorb Eu³⁺, La³⁺, and Sm³⁺ ions was rigorously evaluated. The ideal conditions for lanthanides removal were determined by carefully controlling and optimizing key adsorption factors, including reaction temperature, starting metal concentration, adsorbent mass, contact time, and solution pH. Furthermore, pH 3.5, contact duration of 240.0 min, adsorbent weight of 0.05 g, and starting concentration of 100 mg/L are the optimal conditions for removing La^3+^, Eu^3+^, and Sm^3+^ ions onto ZrO_2_. Kinetic modeling revealed that the results were most accurately represented by the pseudo-second-order model, which provided the most accurate for characterizing the adsorption procedure. The obtained data exhibited a resilient correlation to Langmuir and Freundlih models. The thermodynamic analysis further showed that La^3+^, Eu^3+^, and Sm^3+^ adsorbed spontaneously and endothermically on ZrO_2_. Reusability assessments demonstrated the adsorbent’s efficacy over five regeneration cycles. Thus, the promising affinity of ZrO_2_ for preferential adsorption of La^3+^, Eu^3+^ and Sm^3+^ ions from acidic media is useful.

## Introduction

Lanthanides, yttrium, and scandium are all included in the collective noun known as rare earth elements (REE). Due to the difficulty of separating each element from its corresponding mineral, rare earth compounds are costly. REEs are strategically important for the advancement of contemporary societies. Lanthanum, europium, and samarium are important rare earth elements widely utilized in fields such as metallurgy, electronics, agriculture, natural sciences, ceramics, the manufacturing of glass^1^. Europium, a typical lanthanide element, and it usually exists in the trivalent state (^+3^), while it can also occasionally be found in the divalent state (^+2^)^2^. As homologs of trivalent actinide elements, they have been used because of their comparable physico-chemical characteristics. Around 0.015% of the earth’s crust is made up of lanthanides, which is comparable to that of several heavy metals, including lead, copper, zinc, and arsenic, indicating a similar degree of abundance within the geological composition^3^. In addition, even in trace amounts, lanthanides are present in a wide variety of rocks. The demand for lanthanides chemical elements is continually rising due to their remarkable application in numerous industrial fields.

Lanthanum, the inaugural element of the lanthanides series, demonstrates versatile applicability in the creation of hydrogen storage materials, sophisticated camera lenses, and catalytic converters for automobiles, highlighting its significance in both technological and environmental applications^4^. In the end, samarium (Sm) is employed as a neutron absorber in addition to being utilized in manufacturing specialized optical glasses and modifying ceramic materials. Furthermore, samarium is a key component in the creation of high-performance samarium-cobalt magnets, renowned for their strong magnetic fields. It contains seven isotopes in nature as well; three have a lengthy half-life, while four are stable. Monazite, bastnasite, and xenotime are examples of key independent rare earth minerals, the main types of primary ores commonly used for extracting rare earth elements (REEs)^5^. REEs are generally found at characteristically low concentrations within native ore deposits, typically occurring at trace levels measured in milligrams per kilogram. This scarcity necessitates extensive processing and separation techniques for REE extraction and subsequent utilization in various industrial applications^6^. Furthermore, possible sources for rare earth extraction include phosphorites, bauxites, deep-sea sediments, and tailings (such as phosphogypsum and red mud)^7^. Throughout the entire rare earth production process, substantial amounts of waste that consists of varying concentrations of rare earth elements. This phenomenon is observed during mining, beneficiation, separation, smelting, and further processing stages, irrespective of the source ore’s composition^8,9^. Ln(III) and numerous recyclable actinides (An(III)) made up the spent fuel. The essential components for commercially viable secondary sources can be recovered and extracted from these recyclable solid wastes^1,10^. However, due to the global social impact, radioactive spent fuel management is receiving more attention. As the nuclear fuel cycle progresses, nuclear fission and a large number of neutron capture events a significant quantity of lanthanide and actinide elements present. Lanthanides are key fission product isotopes formed during the irradiation of nuclear fuel. Environmental assessments for dumped long-lived radioactive waste have shown a considerable deal of interest in the environmental behavior of lanthanides^11^. For long-term waste management, it is therefore highly beneficial to separate these components effectively before geological burial. In terms of the transmutation approach for managing long-lived actinide emitters, it would be particularly crucial to separate neutron-absorbable lanthanides.

The harmful impacts of REEs on human health are comparable to those of heavy metals. Because different technologies generate several million tons of waste solutions containing rare earth elements annually, preconcentration, recycling, and the extraction of REEs from these wastes remain unresolved issues^12^. Nearly all lanthanides have stable (^+3^) oxidation states and identical ionic radii, which make their physico-chemical characteristics a significant barrier to their recovery and separation^13^. Preconcentration and separation of REEs can be employed using a various techniques including solvent extraction, ion exchange, membranes, adsorption and precipitation. Unfortunately, most of these methods have a number of drawbacks, including high operating costs, excessive reagent consumption, ineffective pollutant removal, secondary pollutant production, etc. Adsorption is an amazing approach known for its low cost, excellent process efficiency across a wide concentration range, no harmful by-products, simplicity, and minimal environmental impact. Adsorption may be used for efficient extraction of lanthanides over wide range of concentrations aqueous media. However, the adsorption efficiency is basically based on the chosen adsorbents and their functionalization. Moreover, various adsorbents are employed for separation or extraction of REEs from aqueous solutions. Numerous studies have shown that nanotechnology can be used effectively in the remediation of metal contamination. This involves the removal of pollutants using carbon nanotubes, bioactive nanoparticles, nanocatalysts, nanostructured catalytic membranes, nanosorbents, and molecularly imprinted polymers (MIP)^14^. A thorough plan for lanthanide recovery in the fields of nuclear engineering or natural resource development will be provided by the efficient extraction or separation of lanthanides from various solutions^1^. Environmental authorization protection groups have recently advocated for and legislated green chemistry. The European Union, the US EPA, and numerous other organizations have pushed scientists to find and create greener compounds using conventional methods and technologies. So, finding and designing more environmentally friendly nanosorbents or nanocomposites with strong metal ion removal capabilities is an important issue for many environmental and pollution control fields.

The term “green synthesis” or “plant-mediated synthesis” has been developed recently for the synthesis of NPs, which do not require a stabilizing agent or reducing agent and greatly aid in environmental conservation by lowering human risk factors^15^. Researchers are interested in the creation of metal nanoparticles (NPs) because of their many potential uses. Rich in polyphenolic compounds, pomegranate fruit has been demonstrated to have superior antioxidant qualities. Pomegranate fruit is also rich in potassium, oxalic acid, and vitamins A, B6, B9, and E. Pomegranate peel (PP), an agricultural byproduct, makes up over half of the fruit’s total weight. Accordingly, metal oxide nanoparticles have become promising options for treating wastewater on a wide scale in industrial environments. Advantageous features of these nanoparticles include affordability, remarkable adsorption ability, stable physicochemical characteristics, easey separation, and enhanced stability. These characteristics allow nanoparticles to effectively remove metal ions from wastewater^16^ like iron oxide^17^, aluminium oxide^18^, titanium oxide^19^ and manganese oxide^20^. Due to their great mechanical strength, remarkable chemical inertness, and high thermal stability, ZrO₂ nanoparticles have emerged as a multifunctional material^21^. An environmentally friendly, economically viable, and sustainable metal, zirconium oxide widely used in fields like oxygen sensors, fuel cell electrolytes, dentistry^22^, ceramics, and electro-chromic devices^23^, Intracellular imaging^24^, heavy metal sequestration^25^. Zirconium oxide can be produced environmentally by avoiding hazardous reactants, which are typically involved in conventional processes and lead to toxic intermediates^26^. Moreover, plant-mediated phytonanofabrication is a cost-effective process that does not require special production conditions and is simple to manage. This study focuses on creating zirconium oxide (ZrO_2_) using pomegranate peel extract to separate certain lanthanides elements, including La^3+^, Eu^3+^, and Sm^3+^. The novelty herein lies in designing ZrO_2_ using a fully biobased process and their use in the elimination of La^3+^, Eu^3+^, and Sm^3+^ ions from acidic media. Additionally, ZrO_2_ was comprehensively characterized utilizing multiple techniques, including FTIR, TGA-DTA, XRD, and SEM-EDX mapping. To provide more accurate predictions regarding the adsorption mechanism in this context, appropriate isotherm and kinetic models were applied. To set up application, ZrO_2_’s reusability and regenerative qualities were thoroughly examined.

## Materials and methods

### Chemicals

Every chemical employed within this investigation was of analytically grade and was not further sanitized before use. The following chemicals were exploited in the current work: LaCl_3_.6H_2_O, SmCl_3_.6H_2_O, HNO_3_ and EuCl_3_. 6H_2_O were obtained from MERCH, Germany. As well, ZrOCl_2_.8H_2_O was supplied by Sigma-Aldrich. Besides, HCl, and NaOH were purchased from adwic, Egypt.

### Pomegranate peel extract preparation

The local Egyptian market provided the fresh pomegranate fruits that were needed. After being separated from the fruit, the peels were subjected to a thorough water rinse, followed by a drying step in a 60 °C oven. A home blender used to grind the dried peels into a powder. Appropriate amount of powder was added to hot water at 50 °C for 4 h. After cooling, the solution was clarified to have pure pomegranate peel extract and it used for further synthesis.

### Synthesis of ZrO_2_ particles by pomegranate peels extract

The technique of preparing ZrO_2_ particles involves several steps. Initially, pomegranate peel extract was prepared. Second, 200 ml of ZrOCl_2_·8H_2_O (0.1 M) was mixed with 300 ml of freshly prepared pure pomegranate peel extract, and the mixture was constantly agitated for four hours at 70 °C. A 0.1 M NaOH solution was added dropwise to the reaction mixture under constant stirring for 2 h until the pH reached approximately 9–10, leading to the formation of a white gelatinous precipitate of zirconium hydroxide. Subsequently, the mixture needs to be set for centrifugation at 4000 rpm for 14 min to eliminate the leftover organic molecules and extra ZrOCl_2_. The residue was washed before being dried at 70 °C for 2 h. Ultimately, zirconium oxide particles (ZrO_2_) must undergo a furnace calcination process, which entails heating at 400 °C for two hours.

### Characterization

Herein, ZrO_2_ morphology was examined by SEM-EDX (XL30SFEG-TMP, Phillips, Netherlands). The FTIR spectra of ZrO_2_ was exploited by KBr method on fourier transformed infrared spectroscopy, a BOMEM FTIR model MB 147, Canada, Nicolet, USA. To perform thermogravimetric and differential thermal analyses (TGA-DTA), a Shimadzu DTA-60 thermal analyzer (Kyoto, Japan) was exploited. Moreover, to determine its crystal structure, X-ray diffractometer was employed (Shimadzu Model XD 490, Kyoto, Japan). Additionally, a Shimadzu UV-visible spectrophotometer (Japan) employed to quantify metal ions concentrations. A thermostate shaker, manufactured in Clifton, England, was used to shake the solid-liquid phases. A microprocessor-equipped digital pH meter (model AD/030, Romania) was working to measure the metal ion solutions pH. All of the materials and samples used in this experiment were evaluated by Ae ADAM PW124 analytical balance (Germany) with 120 g of maximum capacity and a precision of 0.0001 g. An oven (Binder, FD 53, USA) was used to calcine and dry materials.

### Preparation of stock solutions

The respective chloride salts of La^3+^, Eu^3+^, and Sm^3+^ ions dissolved in double-distilled water to prepare standard solutions with precisely known concentrations. Furthermore, various concentrations of employed metal ions (100, 200, 400, 600, and 800 mg/L) were created by suitably diluting stock solutions.

### Batch experiments

To quantify the adsorption performance of ZrO₂, its capacity for La^3+^, Eu^3+^, and Sm^3+^ ions was measured via multiple batch experiments. The impact of starting metal ion concentrations (100.0-800.0 mg/L), adsorbent mass (0.01–0.125 g), pH values (1.0–5.0), adsorption time (5.0–240.0 min) and temperature (298–338 K) on the removal efficiency were investigated by changing one variable but others remained almost constant. The pH was controlled through the drop wise subtraction of 0.01 M HCl or NaOH as required. For experimental tests, In 100 ml flasks, batch investigations were evaluated to assess how solution pH influences the adsorption efficiency of ZrO_2_. The investigation involved varying the pH between 1.0 and 5.0, with the remaining variables kept unchanged. Furthermore, the adsorbent dose studies were carried out at various dose levels (0.01–0.125 g) while keeping a constant pH of 3.5, starting concentration (100 mg/L), and contact period of 4 h. The mixture was subsequently primed in a temperature-regulated shaker bath, where it was continuously agitated at 400 rpm over a 4-hour period. After filtering the mixtures, the resulting filtrates were then analyzed for La^3+^, Eu^3+^, and Sm^3+^ ions content with UV-visible spectrophotometry by Arsenazo(III) as the complexing reagent. The removal percent (%R) was measured as follows:

1 $$\% R=\frac{{{C_o} - {C_e}}}{{{C_o}}}x100$$

In this formula, C_o_ and C_e_ correspond to the metal ion concentration under study at the start of the process and at equilibrium, respectively, (mg/L).

### Adsorption kinetic studies

Using 100 ml flasks, tests were conducted to examine the kinetic adsorption process. A solution of La^3+^, Eu^3+^, and Sm^3+^ ions containing (100 mg/L) was mixed with 0.05 g of ZrO_2_ at pH 3.5 and 25 °C. At 400 rpm, the mixture was agitated on a thermal shaker. After every adsorption cycle, the supernatant solution filtrate was then analyzed to determine the equilibrium metal ion concentration. The kinetics of La^3+^, Eu^3+^, and Sm^3+^ adsorption by ZrO_2_ were analyzed using the pseudo-first-order, pseudo-second-order, Elovich, and intra-particle diffusion models. These equations were described by the following:

2 $$\log ({q_e} - {q_t})=\log {q_e} - \frac{{{k_1}}}{{2.303}}t$$

3 $$\frac{t}{{{q_t}}}=\frac{1}{{{K_2}{q_e}^{2}}}+\frac{t}{{{q_e}}}$$

4 $${q_t}=\beta \ln (\alpha \beta )+\beta \ln t$$

5 $${q_t}={K_i}{t^{0.5}}+C$$

Herein, q_e_ and q_t_ stand for the amount of metal ions adsorbed, in mg per gram of adsorbent, at equilibrium and at any time *t*, respectively. Whereas K_1_ (min^− 1^) represents the rate constant. While k_i_ is the intra-particle diffusion rate constant (mg/g·min^− 1^). In contrast, the pseudo-second-order rate constant (K_2_) is expressed in (mg/g.min). Furthermore, β denotes the activation energy necessary for the chemisorption process, while α signifies the initial adsorption rate, expressed in mg/g·min.

### Adsorption isotherms

Adsorption isotherm studies were conducted using batch experiments. The study analyzed the sorption potential of ZrO_2_ for removing La^3+^, Eu^3+^, and Sm^3+^ ions from aqueous solutions. For each assay, a 100 ml solution containing La^3+^, Eu^3+^, and Sm^3+^ ions at a concentration of 100.0–800.0 mg/L was combined with 0.05 g of ZrO_2_ in an Erlenmeyer flask. After four hours of shaking, the samples were filtered and analyzed to determine La^3+^, Eu^3+^, and Sm^3+^ ions concentrations. The adsorption behavior was characterized by modeling the isotherms with the Langmuir, Freundlich, Dubinin–Radushkevich, and Temkin equations (shown below):

6 $$\frac{{{C_e}}}{{{q_e}}}=\frac{1}{{{q_o}b}}+\frac{1}{{{q_o}}}{C_e}$$

7 $${R_L}=\frac{1}{{1+b{C_o}}}$$

8 $$\log {q_e}=\log {K_f}+\frac{1}{n}\log {C_e}$$

9 $$\ln {q_e}=\ln {Q_m} - K{\varepsilon ^2}$$

10 $$\varepsilon ={R_g}T\ln \left( {1+\frac{1}{{{C_e}}}} \right)$$

11 $$E={( - 2K)^{ - 0.5}}$$

12 $${q_e}=BLn({A_T})+BLn({C_e})$$

### Competitive adsorption

To examine competitive behavior and efficiency, adsorption studies of La^3+^, Eu^3+^, and Sm^3+^ ions were carried out with co-existing ions such as Cs^+^, Sr^2+^, and Co^2+^ to show their competitive interactions and adsorption efficiencies. The investigations occurred under pH of 3.5, at room temperature.

### Desorption investigations and adsorbent reusability

In this study, lanthanum-loaded ZrO_2_ was subjected to desorption studies using various solutions to determine their effectiveness in releasing La^3+^, Eu^3+^, and Sm^3+^ ions. A suspension containing ZrO_2_ (100 mg) and La^3+^, Eu^3+^, and Sm^3+^ ions solution (10 ml of 100 mg/L) was shaken at 400 rpm for four hours. Following lanthanum-loaded ZrO_2_, the material underwent treatment with 10 ml aliquots of hydrochloric acid, nitric acid, sodium hydroxide, or ethylenediaminetetraacetic acid. Consequent, the solids were meticulously extracted from the solutions that had been gathered for analysis. Spectrophotometric measurements were conducted utilizing UV–Vis spectrophotometer for determining La^3+^, Eu^3+^, and Sm^3+^ ions concentrations^27^. Also, the desorption percentage was calculated.

Recycling the spent adsorbent allowed for the evaluation of ZrO_2_’s economic viability and reusability. In a standard run, 0.1 g ZrO₂ was combined with La^3+^ solution (10 ml, 100 mg/L) and shaken for a duration of 4 h to achieve equilibrium. Besides, La^3+^-adsorbed ZrO₂ was also treated by soaking in 10 ml of 0.1 M hydrochloric acid. This suspension was then agitated for 4 h at ambient temperature. Following each treatment, ZrO_2_ was carefully separated from the La^3+^ solutions. Subsequently, it underwent a rinsing process using distilled water to remove any residual contaminants. After filtration, the solid residue was resuspended in a 10 ml solution of freshly prepared (100 mg/L) La^3+^, and subsequently agitated. Following each cycle, encompassing a maximum of five consecutive iterations, the efficacy of La^3+^ removal was rigorously achieved.

## Results and discussions

### Analysis of the synthesized materials

**Fig. 1:**
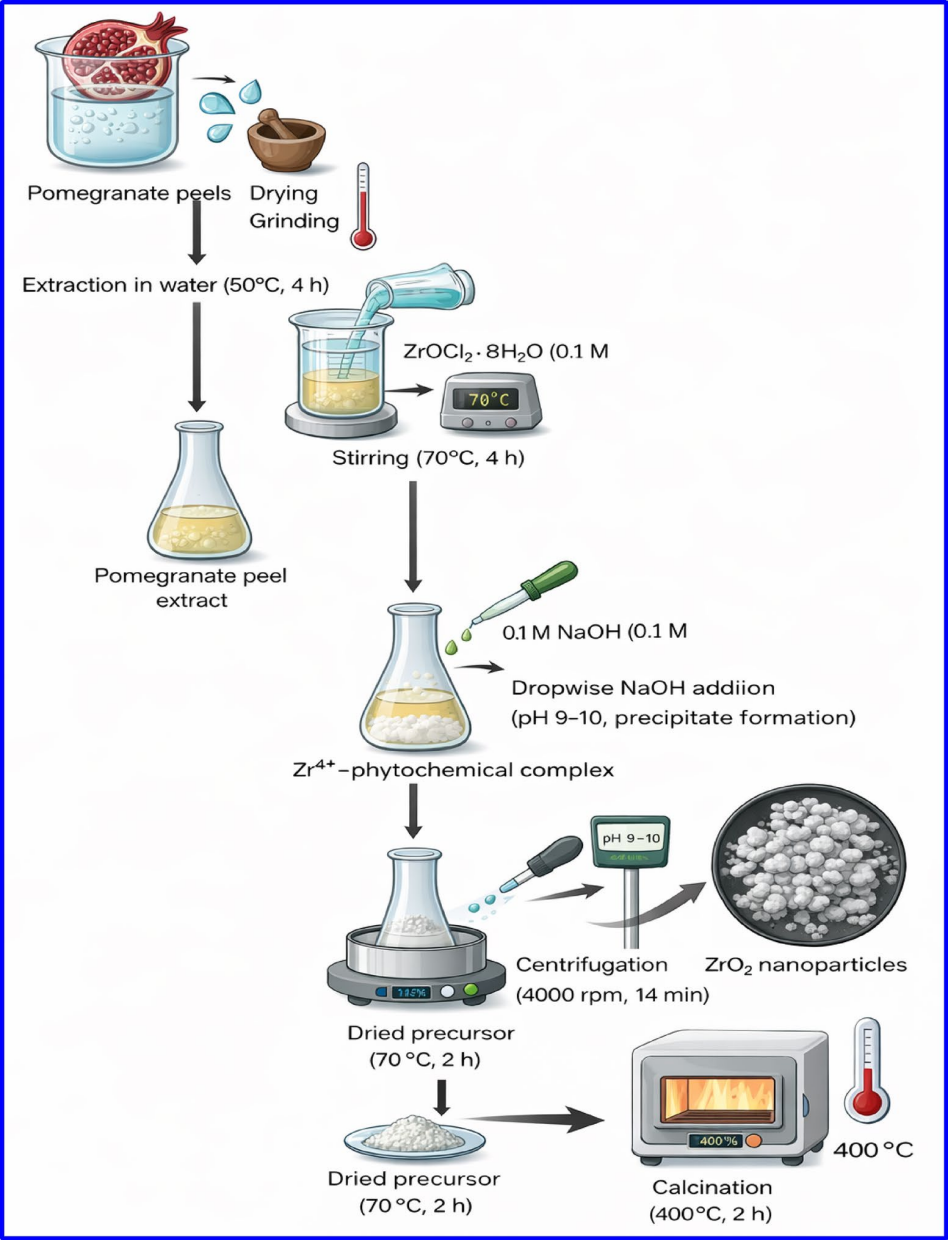
Schematic representation for ZrO_2_ preparation by pomegrante peel extract.

In this study, a comprehensive characterization approaches, including FTIR, XRD, SEM–EDX mapping, and TGA–DTA, were applied in order to confirm the successful preparation of ZrO_2_. Figure 1 showed the schematic representation of ZrO_2_ preparation method.

### FTIR study

**Fig. 2:**
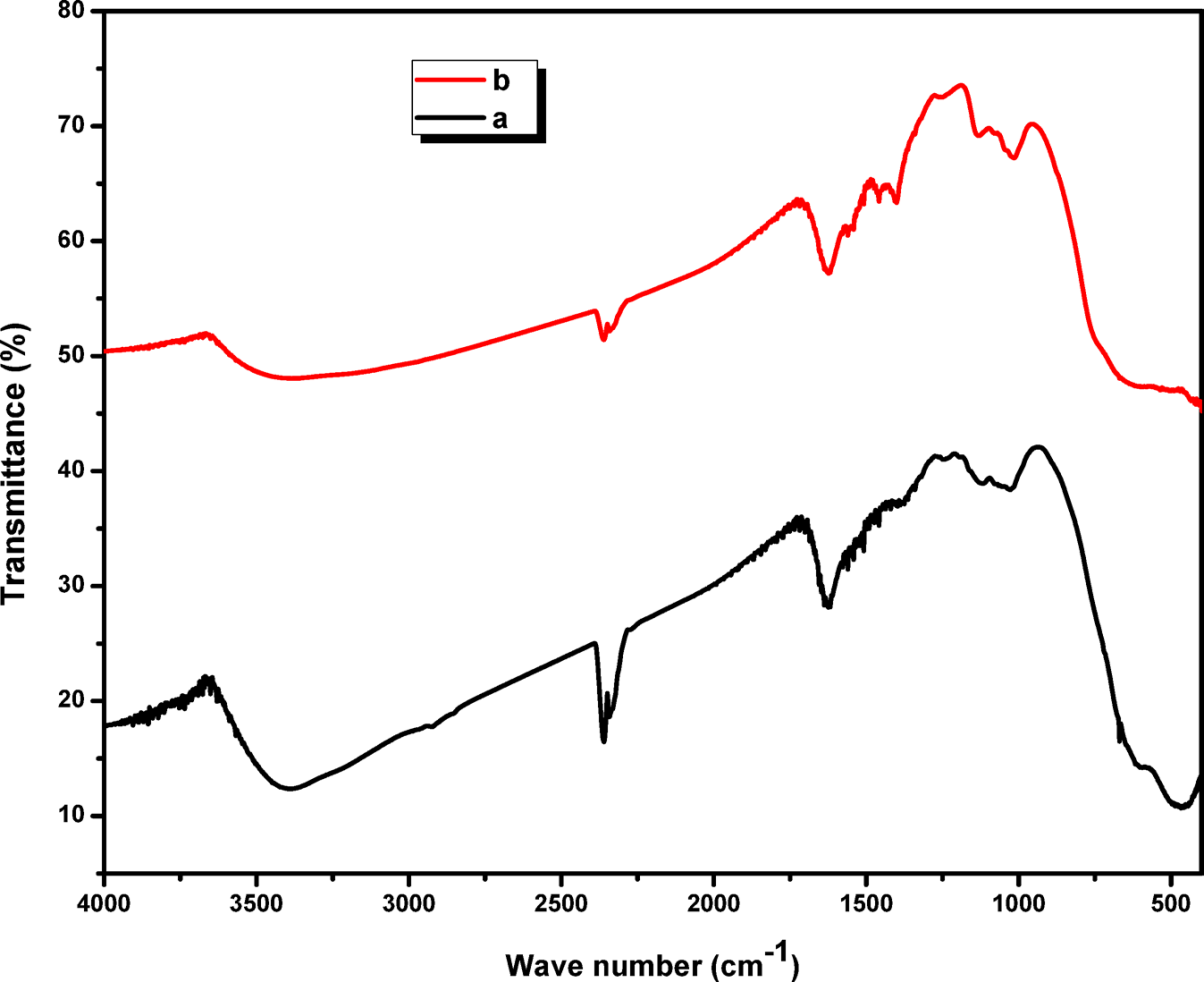
(a) FTIR spectrum of ZrO_2_ before adsorption process (b) FTIR spectrum of La^3+^- loaded ZrO_2_.

The FT-IR spectrum of the prepared ZrO_2_ sample before adsorption process, provided in Fig. 2a, was used to identify its surface functional groups. Characteristic bands for adsorbed water are evident in the FT-IR spectrum: a broad O–H stretching vibration at 3180 cm⁻¹ and a sharp peak of H–O–H bending vibration at 1655 cm⁻¹. The diminished band strength is probably due to dehydration of internal water molecules, which occurred as a consequence of the higher calcination temperature during synthesis. Consistent with the work^28^, the peaks assigned at 664 and 474 cm⁻¹ to Zr–O bond vibrations. After adsorption, as shown in spectrum (Fig. 2b), noticeable shifts and intensity changes occur in these bands, particularly the hydroxyl and Zr–O stretching regions, indicating coordination or electrostatic interaction between La(III) ions and the oxygen-containing sites on the ZrO₂ surface. These spectral modifications suggest that La(III) ions are chemically bound to the adsorbent surface, supporting the occurrence of a chemisorption mechanism.

### XRD

**Fig. 3:**
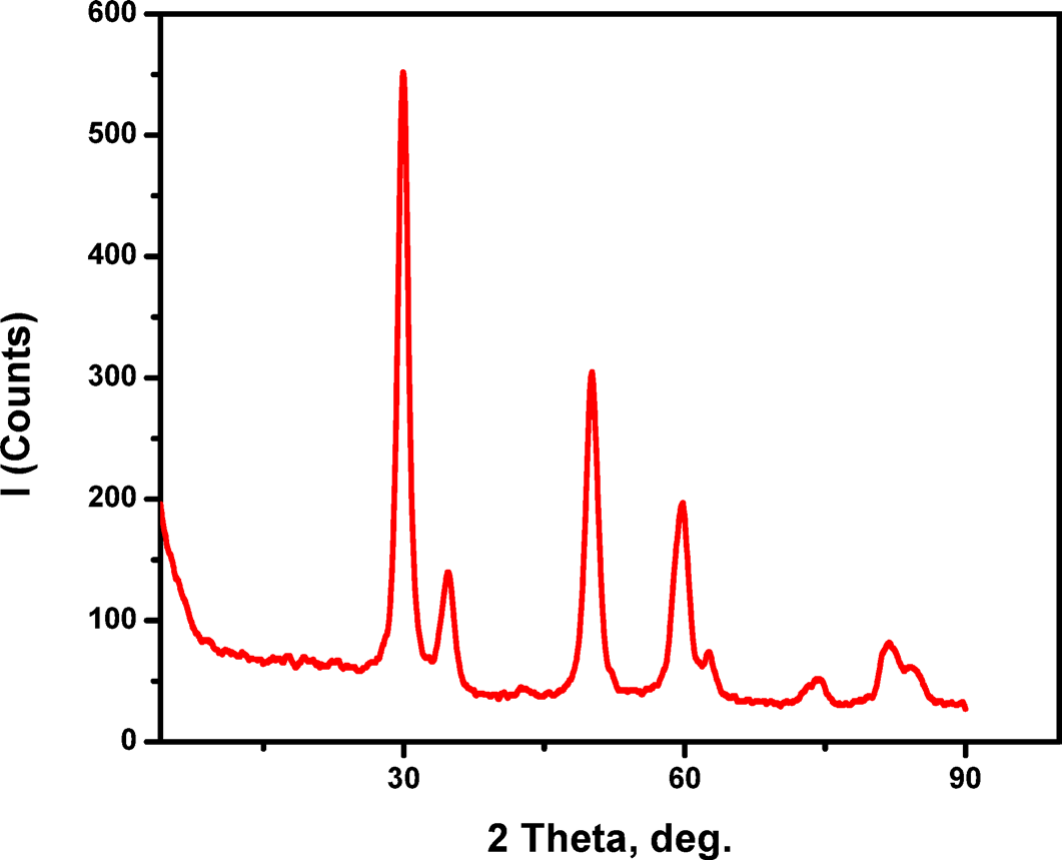
XRD pattern of ZrO_2_ synthesized by pomegranate peel extract.

The crystal structure of ZrO_2_ was evaluated and the XRD profile is displayed in Fig. 3. The crystal structure of monoclinic zirconium oxide was closely matched to XRD pattern of the prepared ZrO_2_. There are five peaks appeared at 2θ = 28.6, 33.1, 47.36, 56.19, and 69.01^o^. The observed peaks matched the Miller index values (111), (002), (022), (031), and (131), confirming the crystallographic planes. Consequently, the XRD analysis corroborated that the synthesized nanoparticles manifested a monoclinic crystalline structure^29^.

An approximate crystallite size was estimated from the most intense XRD peak using the Scherrer equation:

13 $$\:\mathrm{D}=\frac{\mathrm{K}{\uplambda\:}}{{\upbeta\:}\:\mathrm{C}\mathrm{o}\mathrm{s}{\uptheta\:}}$$

where D is the crystallite size, K is the shape factor (0.9), λ is the X-ray wavelength (0.15406 nm for Cu Kα radiation), β is the full width at half maximum (FWHM, in radians), and θ is the Bragg angle.Thus, the average crystallite size of ZrO_2_ is estimated to be approximately 14 nm.

### TGA-DTA

**Fig. 4:**
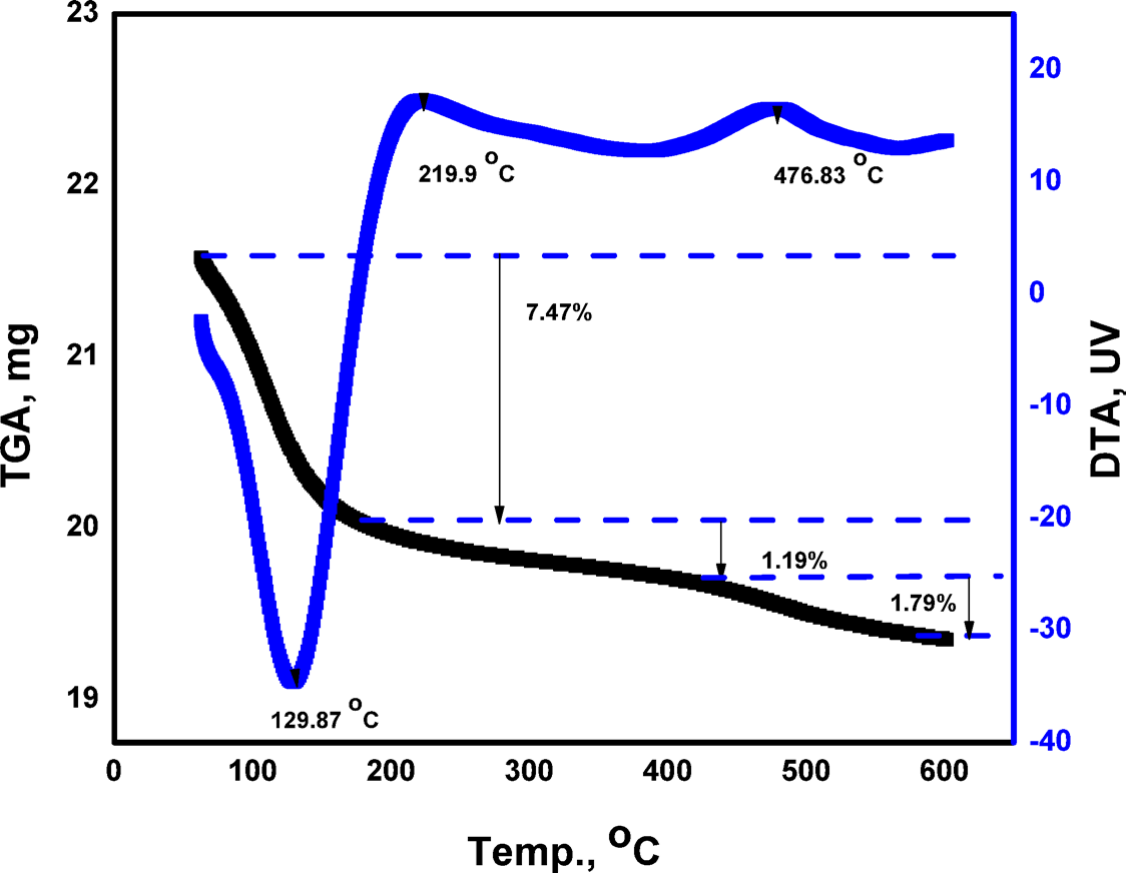
TGA-DTA curves of ZrO_2_.

Figure 4 shows the TGA–DTA curves for the synthesized ZrO₂, characterizing its thermal behavior. The mass loss trend can be seen in two degradation steps. The TGA curve revealed an initial weight loss of approximately 7.47% between room temperature and 200 °C, ascribed to the evaporation of external moisture. The endothermic peak observed at 129 °C on the DTA curve corresponds to the material’s dehydration. There are small continuous degradation steps with small weight losses of 1.19% and 1.79% observed from 200 to 600 °C, matching to two exothermic peaks of DTA curve at 219 and 476 °C. No further mass loss was observed at temperatures above 600 ◦C for ZrO_2_, indicating its stability at higher temperatures^30^. In the 200–600 °C range, the framework of ZrO_2_ remains largely intact, and no significant decomposition of the inorganic lattice occurs. Any minor mass change in this region is typically related to the gradual removal of strongly bound surface hydroxyl groups, residual organic moieties from synthesis, or dehydroxylation processes, which occur slowly and contribute minimally to overall weight loss. Moreover, zirconia is known for its excellent thermal and chemical stability, which explains the absence of pronounced mass loss at elevated temperatures. Therefore, the negligible weight loss in this temperature interval confirms the structural stability and successful formation of thermally stable ZrO_2_, making it suitable for high-temperature and adsorption-related applications.

### SEM analysis

**Fig. 5:**
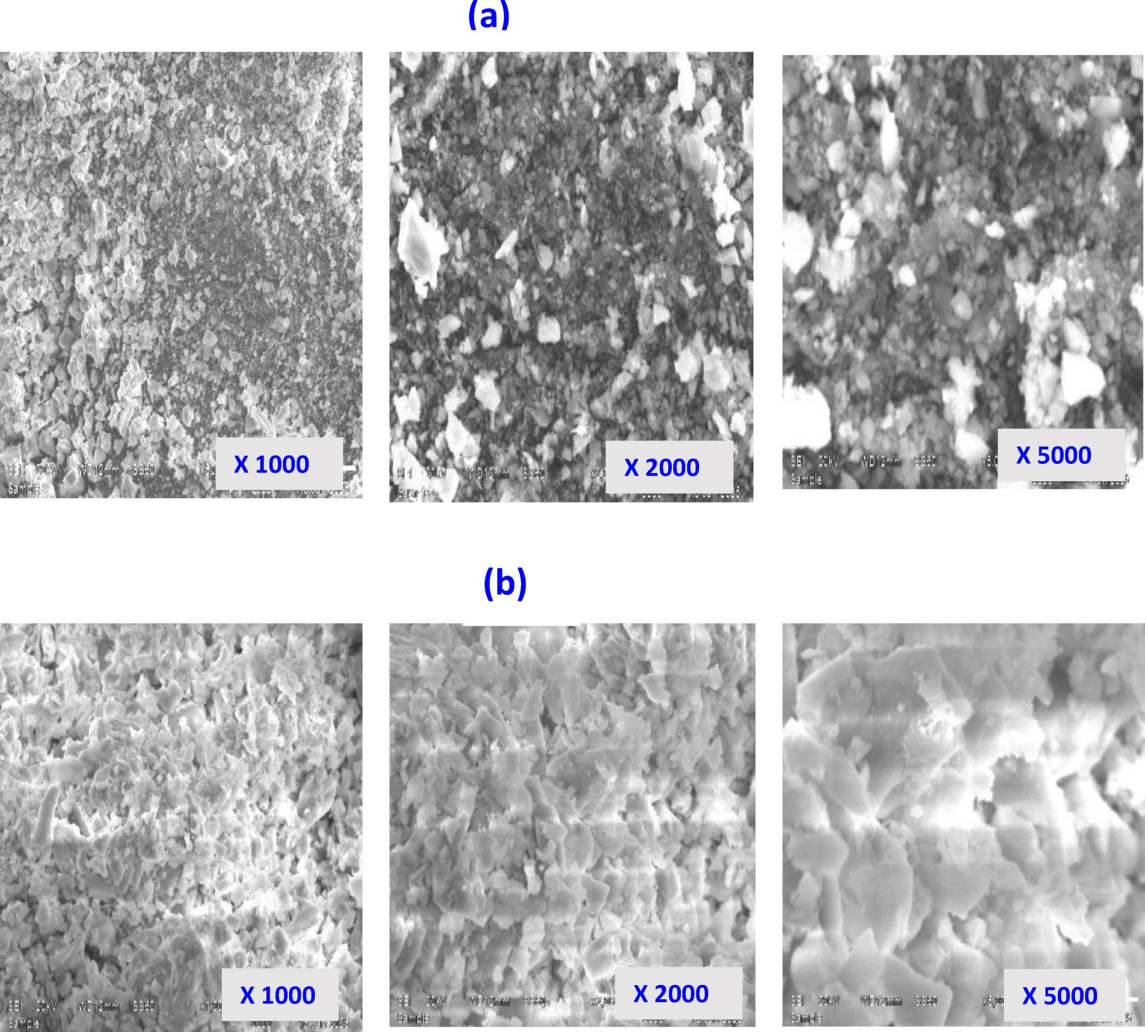
Scanning electron microscope (SEM) images of (**a**) the as-prepared ZrO_2_ before adsorption process and (**b**) after La^3+^ adsorption at different magnifications of 1000, 2000 and 5000 KX.

To investigate the surface morphology and particle geometry, scanning electron microscopy (SEM) was employed^31^. SEM micrographs in Fig. 5a,b compare the morphological features of ZrO_2_ both prior to and after La³⁺ ion adsorption. It can be noticed that SEM images of ZrO_2_ consists of distinct crystalline grains, potential porous regions and discrete particles. Figure 5b shows La³⁺-loaded ZrO_2_ which illustrates that La³⁺ uniformly loaded on the surface of ZrO_2_, as seen by the vibrant elemental signal spots. On the other hand, the SEM picture of ZrO_2_ following the adsorption of La³⁺ seems highly irregular and rough, possibly indicating a fractured surface. Moreover, La³⁺ ions penetrate the pores and bind with functional groups, resulting in a decreased density of gaps and cavities within the material.

**Fig. 6:**
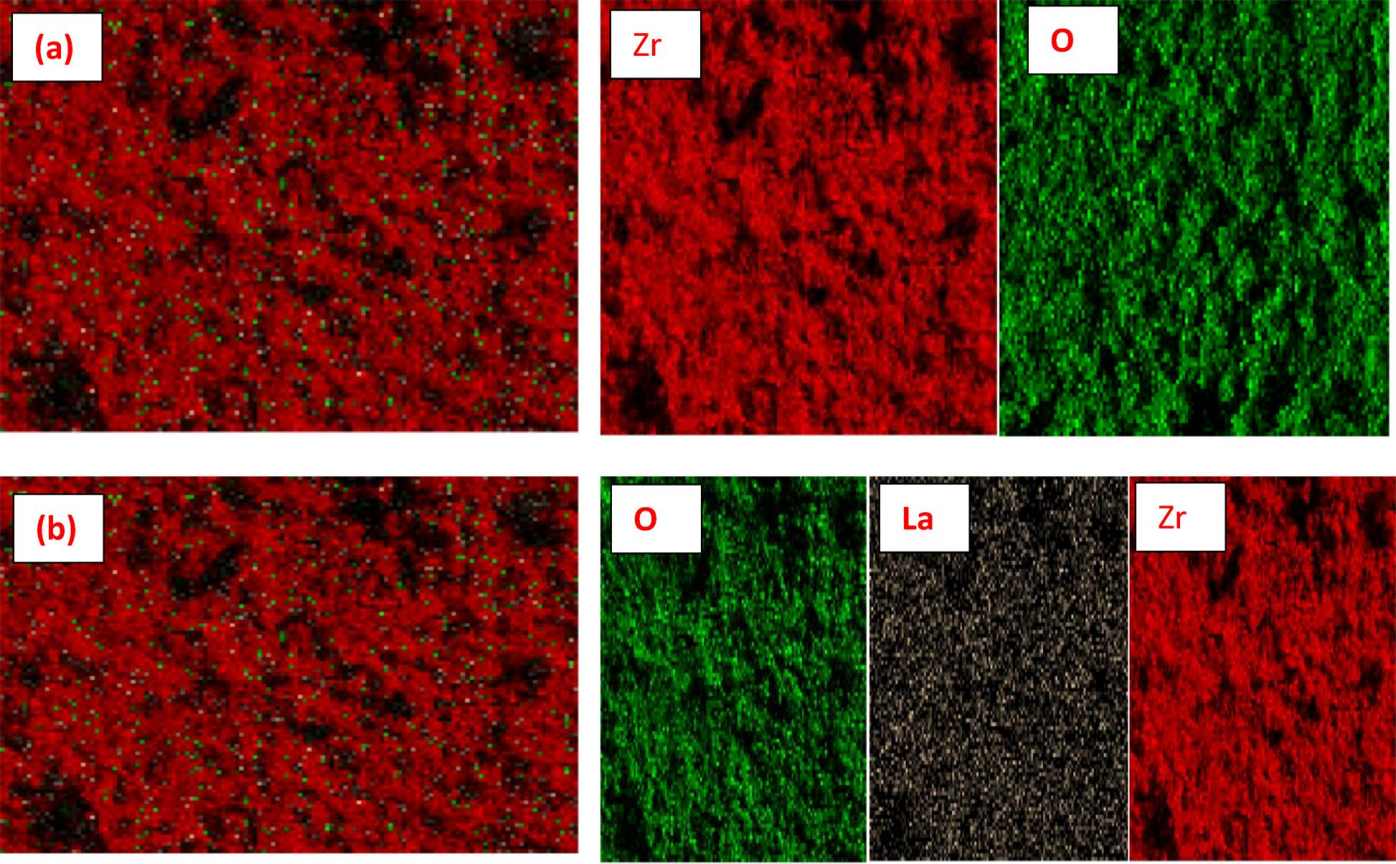
SEM mapping of ZrO_2_ (**a**) before adsorption process and (**b**) after adsorption of La^3+^ ions.

Figure 6 SEM elemental mapping of ZrO₂ (a) before the adsorption process and (b) after the adsorption of La^3+^ ions onto ZrO_2_. The mapping images illustrate the surface distribution of elements, providing insight into the adsorption behavior of La^3+^ ions onto the ZrO₂ surface. In Fig. 6a, the pristine ZrO₂ sample shows a uniform distribution of zirconium and oxygen elements, confirming the purity and homogeneity of the adsorbent surface. After adsorption, as shown in Fig. 6b, new bright spots corresponding to La signals appear, indicating the successful deposition of La^3+^ ions onto the ZrO₂ surface. The increased elemental intensity of La^3+^ ions and its even dispersion across the surface suggest strong interaction and efficient binding between La^3+^ ions and the active sites of ZrO₂, further supporting the chemisorption mechanism inferred from the FTIR analysis.

**Fig. 7:**
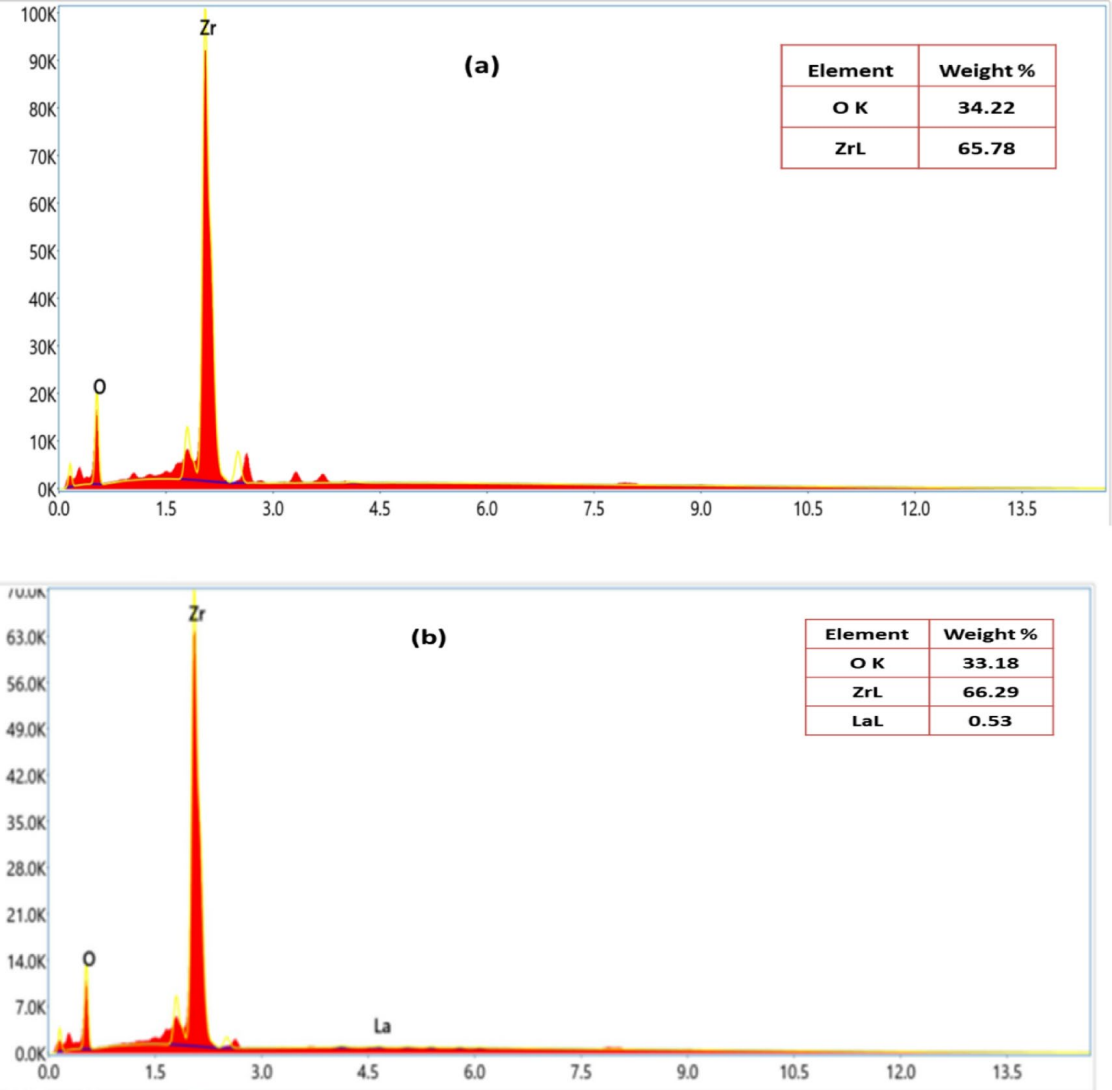
EDX mapping of ZrO_2_ (**a**) before adsorption process and (**b**) after adsorption of La^3+^ ions.

Figure 7a,b illustrates the elemental composition of ZrO_2_ prior and following to La^3+^ adsorption, obtained using EDX mapping analysis. Figure 7a displays the EDX chart of ZrO_2_ before La^3+^ adsorption that illustrates the presence of distinct Zr and O peaks indicative. The elemental composition was determined to be 65.78% zirconium and 34.22% oxygen, indicating the relative abundance of these constituents within the sample. The EDX chart of La^3+^ loaded ZrO_2_ in Fig. 7b shows the detection of La, Zr and O and exhibite a discernible reduction in the percentages of zirconium and oxygen. This decrease correlates with the pronounced presence of La^3+^, indicating successful lanthanum incorporation onto the ZrO_2_ adsorbent surface.

### Effect of solution pH

**Fig. 8:**
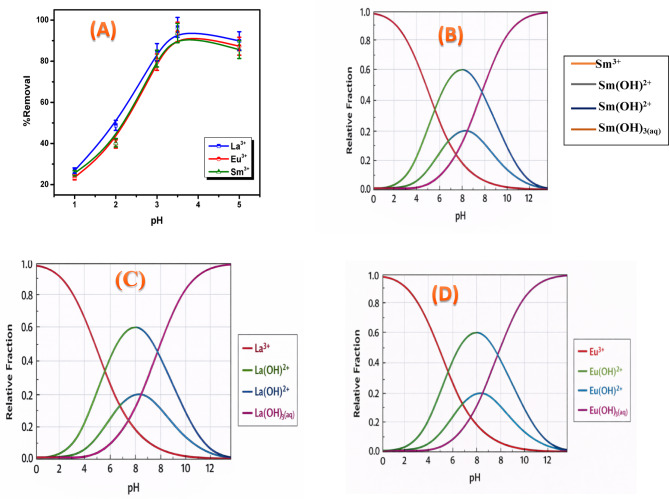

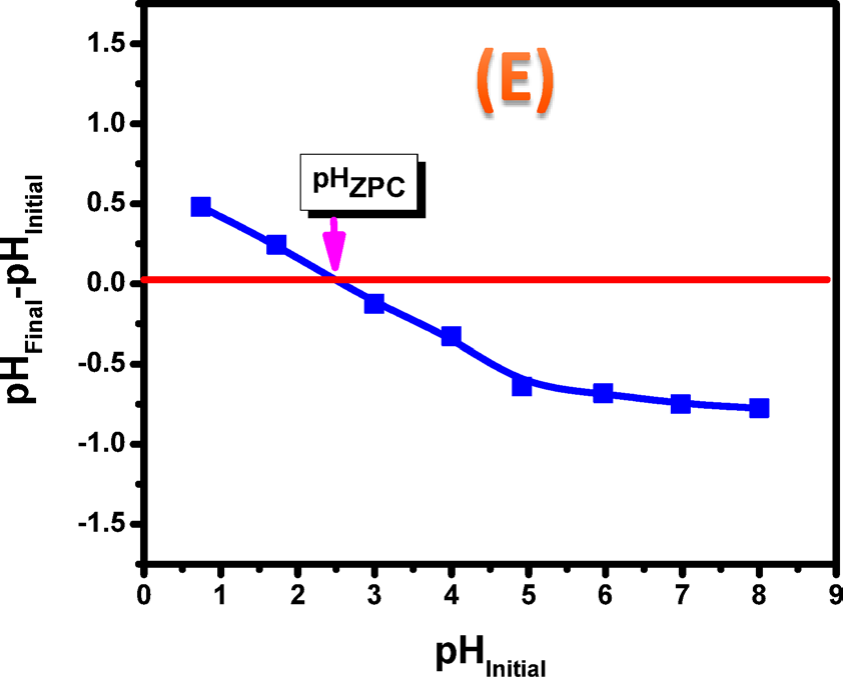
(**A**) Effect of solution pH on removal percent of La^3+^, Eu^3+^ and Sm^3+^ ions onto ZrO_2_ (C_o_,100 mg/L; m, 0.05 g; t, 4 h; T, 25 °C), (**B**) speciation diagram of Sm^3+^, (**C**) speciation diagram of La^3+^, (**D**) speciation diagram of Eu^3+^ and (**E**) Zero point charge of ZrO_2_.

Several factors affect the adsorption performance of materials, with solution pH being one of the most critical parameters. Solution pH critically influences adsorption by concurrently governing the adsorbent’s surface charge and the metal ion speciation. This relationship underscores the importance of pH control for optimizing adsorption processes. The role of solution pH in the removal of La^3+^, Eu^3+^, and Sm^3+^ ions with a ZrO₂ was systematically evaluated. A systematic analysis of adsorption under assorted pH values was done to establish the removal efficiency of (REEs) at the optimal adsorption conditions. In each experiment, 50 mg of ZrO₂ was combined with a solution of (La^3+^, Eu^3+^, and Sm^3+^ ions) (100 mg/L). The pH was attuned to a range of 1.0–5.0, and the suspensions were then agitated at 400 rpm at 25 °C. However, the metal ion removal percentages were plotted against solution pH in Fig. 8A. At pH 3.5, ZrO_2_ exhibited significant adsorption capabilities for La^3+^, Eu^3+^, and Sm^3+^ ions of 96.48%, 94.26%, and 93.71%, respectively. The pH range investigated was limited to 1.0–5.0, as adsorption above pH 6 was not taken into consideration because precipitation began above this point and insoluble metal hydroxides were formed. By increasing the pH values, the uptake of La^3+^, Eu^3+^, and Sm^3+^ ions by ZrO_2_ was exaggerated progressively as the pH was raised from 1.0 to 4.0., reaching its highest value at pH 3.5. The adsorption trend of La^3+^ is directly correlated with its pH-dependent aqueous speciation^32^.

The pH-dependent speciation of Sm^3+^, La^3+^, and Eu^3+^ ions was also assessed with Hydra/Medusa equilibrium modeling, with the corresponding speciation diagram presented in (Fig. 8B–D). The speciation plots reveal the formation of LaOH²⁺ at about pH 3 and La(OH)₂⁺ at roughly pH 5, whereas the trivalent form of lanthanum (La³⁺) dominates until pH values slightly below 6. When pH is more than 6, La(OH)_3_ prevails and precipitation alone is responsible for the elimination of La^3+^^33^. This trend is interpreted as a consequence of the highly acidic environment. At low pH, a high concentration of H⁺ ions leads to: (1) competitive adsorption between protons and rare-earth ions for active sites, and (2) protonation of the ZrO₂ surface, creating a positive charge that electrostatically repels the cationic La^3+^, Eu^3+^, and Sm^3+^ species. The combined effect of these factors results in lower adsorption efficiency. Nevertheless, higher pH values caused a decrease in H^+^ concentration, which in turn produced more free active sites that were ready for adsorption and facilitating stronger electrostatic interactions with metal ions and thereby improving adsorption. When the pH exceeds 5, the insoluble metal hydroxides begin to form at higher pH levels, which make actual sorption investigations challenging. However, increasing the solution pH beyond 5 complicates accurate sorption studies. The selection of pH 3.5 as the optimum for subsequent experiments in this study stemmed directly from preceding results.

The point of zero charge (pH_p_zc) is a key parameter that defines the pH range over which the surface exhibits charge neutrality and provides insight into the nature of surface active sites as well as the adsorption behavior of the material. As illustrated in Fig. 8E, the pH_p_zc of ZrO_2_ was determined to be 2.7. Under the investigated conditions, the highest adsorption efficiencies were observed at pH 3.5, reaching approximately 96.48, 94.26, and 93.71% for La^3+^, Eu^3+^, and Sm^3+^ ions, respectively.

### Effect of adsorbent dose

**Fig. 9:**
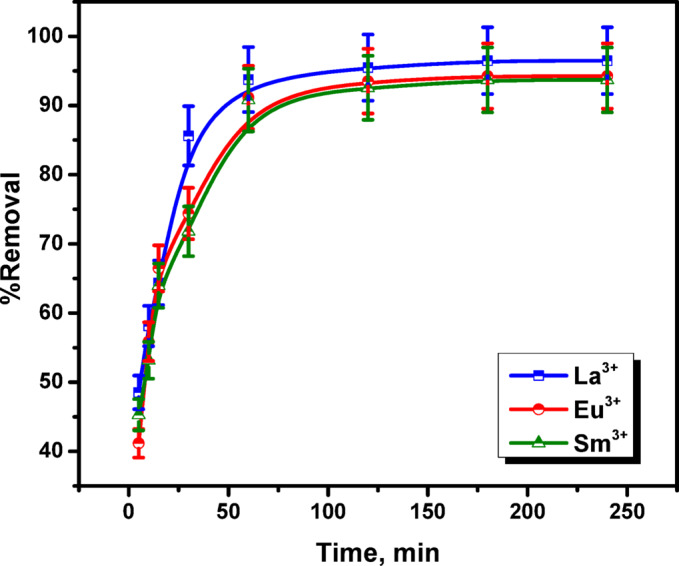
Effect of adsorbent dosage on La^3+^, Eu^3+^ and Sm^3+^ adsorption onto ZrO_2_ (initial metal ions concentration 100 mg/L; pH 3.5; contact time 4 h; temperature 25 °C).

As a fundamental parameter, the adsorbent dosage controls the availability of active sites, thereby directly influencing the adsorption capacity^34^. The effect of ZrO_2_ dosage on the adsorption of La³⁺, Eu³⁺, and Sm³⁺ ions from aqueous solutions was investigated by varying the adsorbent mass from 0.01 to 0.125 g (Fig. 9). The results show that increasing the ZrO₂ dosage from 0.01 to 0.10 g significantly enhanced the removal efficiencies of La³⁺, Eu³⁺, and Sm³⁺ ions from 25.47, 24.87, and 23.87% to 96.48, 94.26, and 93.71%, respectively. This improvement is attributed to the increased availability of surface area and active adsorption sites, facilitating greater metal ion uptake^35^. Beyond 0.10 g, no noticeable increase in removal efficiency was observed, indicating saturation of adsorption sites and possible particle aggregation at higher dosages. Therefore, considering both adsorption efficiency and economic feasibility, 0.05 g of ZrO₂ was selected as the optimum adsorbent dose for subsequent experiments.

### Effect of initial metal ion concentration

**Fig. 10:**
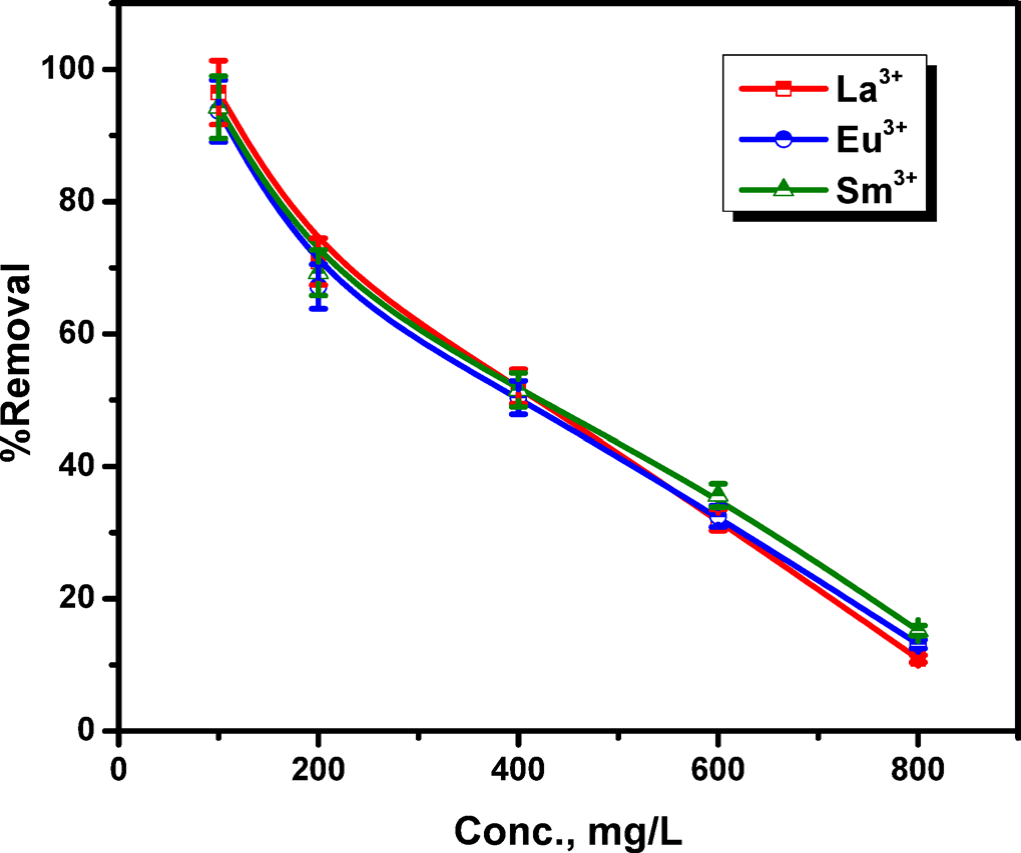
Effect of initial metal ion concentration on La^3+^, Eu^3+^ and Sm^3+^ ions adsorption onto ZrO_2_ (initial metal ions concentration 100–800 mg/L; pH 3.5; contact time 4 h; temperature 25 °C).

The metal ion elimination efficiency is critically dependent on the starting ion concentration. However, La^3+^, Eu^3+^ and Sm^3+^ ions eliminations were examined at a spectrum of starting concentrations, spanning 100–800 mg/L. Figure 10 illustrates the consequence of initial La^3+^, Eu^3+^ and Sm^3+^ ions concentration on the removal efficacy by ZrO_2_. The removal efficiency exhibited a declining trend as the initial metal ion concentration enriched. When the concentration was raised from 100 to 800 mg/L, the adsorption efficiency of ZrO₂ for La^3+^, Eu^3+^ and Sm^3+^ ions declined from (96.48, 93.71and 94.26%) to (15.23, 13.16 and 14.74%), respectively. During metal ion uptake process, high affinity adsorption sites are preferentially occupied when concentrations are low. Nevertheless, at elevated metal ion concentrations, most exchange sites might be occupied, reducing further adsorption^36^.

### Effect of agitation time

**Fig. 11:**
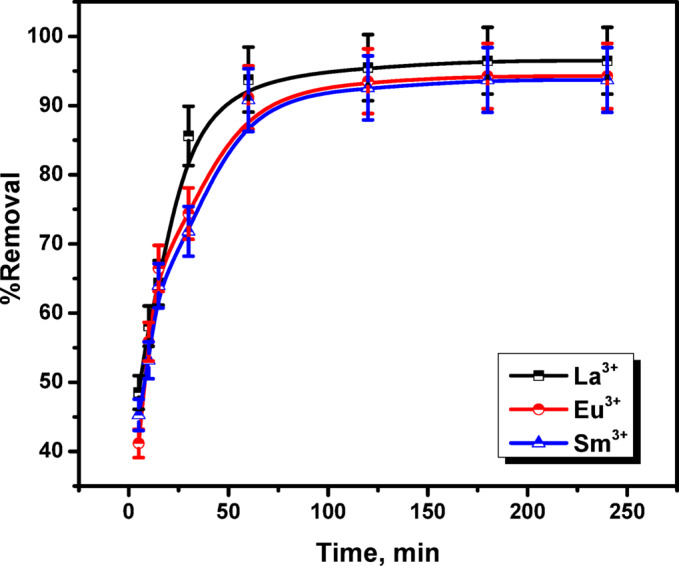
Effect of agitation time on removal percent of La^3+^, Eu^3+^ and Sm^3+^ ions onto ZrO_2_ (initial metal ions concentration 100 mg/L; pH 3.5; adsorbent dosage 0.05 g; temperature 25 °C).

The contact period is mainly crucial constituent in adsorption processes, since equilibrium time is taken into account for demonstrating economic processes. This study aimed to optimize the adsorption time using zirconium dioxide (ZrO_2_), to maximize the removal of La^3+^, Eu^3+^, and Sm^3+^ ions from solution. In Fig. 11, the removal percentage is plotted against contact time, which was varied between 5.0 and 240.0 min. However, ZrO_2_ was found to have removal efficiencies of 93.75, 91.17, and 90.79% for La^3+^, Eu^3+^, and Sm^3+^ ions as a result of quick increase in adsorption rate over the initial 60 min. Afterwards, the adsorption rate stay virtually constant for more than 60 min until equilibrium was achieved since adsorption is not significantly affected by additional increases in contact duration. Since the removal efficiency was marginally improved and the adsorption efficiency increased just little, higher contact time was disregarded, resulting in this scenario being achieved after 240.0 min. Numerous accessible active sites and large adsorbate concentration gradient typical of this early stage may cause the rapid rate of adsorption La^3+^, Eu^3+^, and Sm^3+^ ions by ZrO_2_ within the first 60 min. Afterward, during the adsorption reaction, some unsaturated sites of the adsorbent caused that the removal effectiveness to be gradually increased^37^.

### Adsorption kinetic studies

**Fig. 12:**
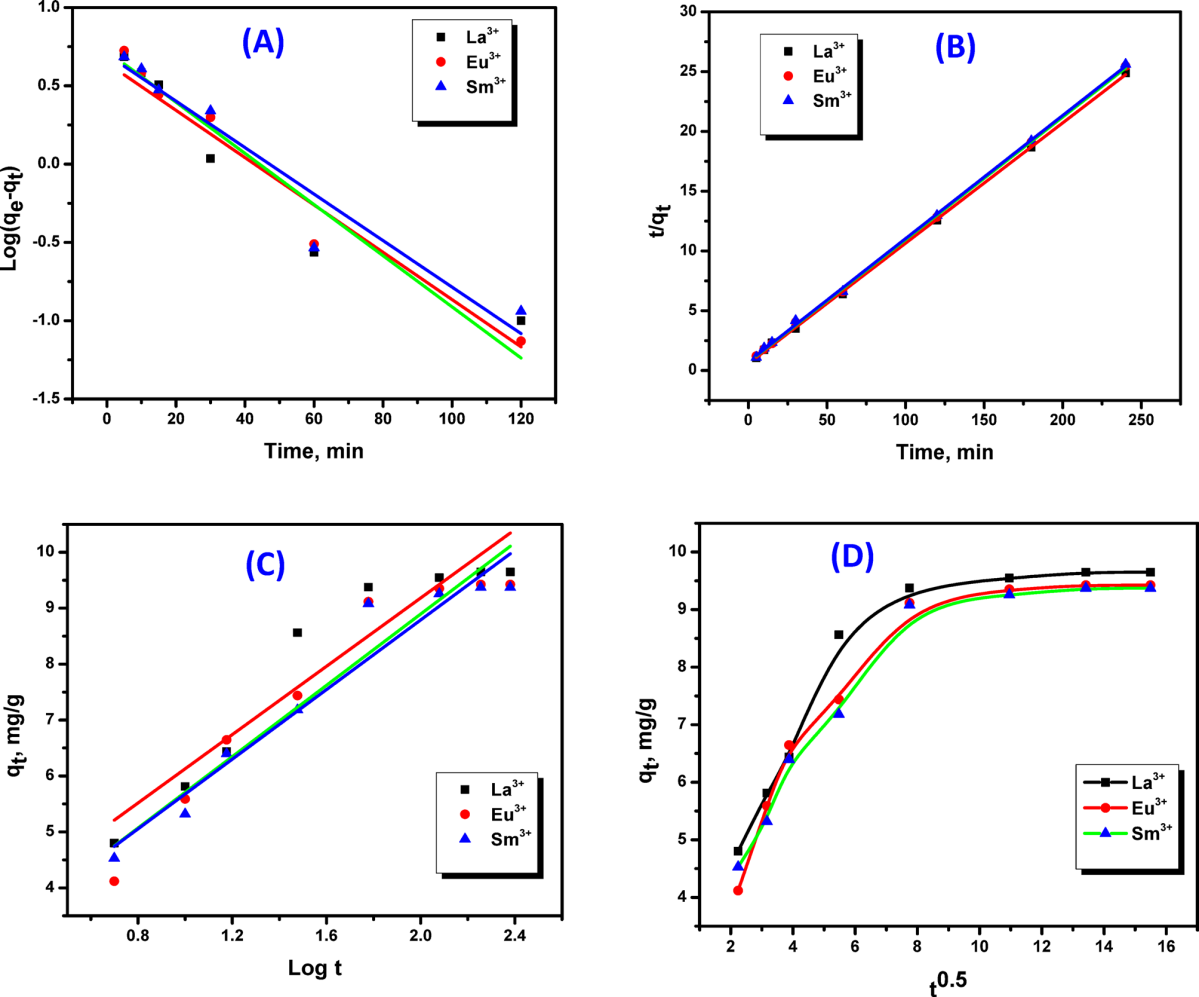
(**A**) Pseudo-first-order plots; (**B**) Pseudo-second-order plots; (**C**) Elovich plots and (**D**) Intra-particel plots for adsorption of La^3+^, Eu^3+^ and Sm^3+^ ions onto ZrO_2_.

Studying adsorption kinetics provides critical insight into the adsorption pathway and further determining the adsorption rate. In this investigation, the kinetics of La^3+^, Eu^3+^, and Sm^3+^ ions uptake on ZrO_2_ were assessed by four kinetic models including pseudo-frst-order^38^, pseudo-second-order^39^, Elovich model and intra-particle diffusion^40^. The experimental data can be analyzed by the above mentioned kinetic models, whereas their plot sets are portrayed in Fig. 12. Additionally, the validation of these models is supported on the index of correlation (R^2^), with the calculated kinetic parameters compiled in Table 1. Considering R^2^ values into account, the sorption of La^3+^, Eu^3+^, and Sm^3+^ ions onto ZrO_2_ is most accurately modeled by pseudo-second-order kinetics (Fig. 12B). Moreover, the (k_2_) for La^3+^, Eu^3+^, and Sm^3+^ ions were 0.017, 0.0149, and 0.014 g·mg⁻¹·min⁻¹, while the corresponding (q_e_) values were 9.93, 9.75, and 9.70 mg/g, and it demonstrated near-perfect correlation (R² = 0.999). The closer agreement between experimental and predicted (q_e_) values highlights that the pseudo-second-order model as more applicable than the pseudo-first-order alternative. Notably, the adsorption of La^3+^, Eu^3+^, and Sm^3+^ ions onto ZrO_2_ is likely dominated by chemisorption, with possible contributions from diffusion-controlled processes.

**Table 1:** Kinetic parameters of PFO, PSO, Elovich and intra-particle diffussion models for the adsorption of La(III), Eu(III) and Sm(III) ions onto ZrO_2_.

Model parameters	Metal ions		
	La(III)	Eu(III)	Sm(III)
Pseudo- first-order model			
k_1_(min^− 1^)	0.034	0.037	0.034
q_e_ ,cal (mg/g)	4.423	5.27	5.004
R^2^	0.918	0.958	0.916
Pseudo-second-order model			
k_2_ (mg g^−1^min^1^)	0.0174	0.0149	0.0143
q_e_ cal. (mg/g)	9.932	9.756	9.708
R^2^	0.999	0.999	0.999
Elovich kinetic model			
α (mg g^−1^min^− 1^)	2.063	1.592	1.685
β (g.mg^− 1^)	1.326	1.384	1.352
R^2^	0.888	0.918	0.931
Intra-particle diffussion model			
K_i_	0.342	0.359	0.356
C	5.311	4.834	4.781
R^2^	0.697	0.734	0.774

The Elovich model is also considered a reliable approach for describing chemisorption behavior. Graphs of (q_e_) against (ln(t)) (Fig. 12C) were employed to evaluate the parameters α and β, as well as the R², are registered in Table 1. The Elovich model revealed that adsorption occurs on heterogeneous surfaces with sites of varying energies and the desorption rate between La^3+^, Eu^3+^, and Sm^3+^ ions and ZrO_2_ surface decreases exponentially as coverage increases^41^. The findings show that the initial adsorption rates (α) of La^3+^, Eu^3+^, and Sm^3+^ ions onto ZrO_2_ were 2.06, 1.59, and 1.66 mg g^− 1^ min^− 1^, respectively. The β parameter, representing the number of adsorption sites, was 1.32, 1.38, and 1.35 g/mg for La^3+^, Eu^3+^, and Sm^3+^ ions, respectively. The strong fit to the Elovich equation, as demonstrated by high R² values, suggests it is an appropriate model for describing the adsorption of these ions onto ZrO_2_. Rendering to the PSO model, these results are consistent with chemisorption process and imply the heterogeneity of adsorbent surfaces.

Kinetic data fitting confirmed that intra-particle diffusion is a relevant mechanism in the adsorption process. A typical method for analyzing the solute transfer during solid-liquid adsorption is the intra-particle diffusion model. To quantify the intra-particle diffusion parameters K_i_ and C for the removal of La^3+^, Eu^3+^, and Sm^3+^ ions by ZrO_2_, q_t_ was plotted against t^1/2^ (Fig. 12D). As per the Weber-Morris model, intra-particle diffusion is confirmed as the dominant mechanism only when the q_t_ vs. t^1/2^ graph pretending a linear trend with an intercept at the origin.

In this study, the intra-particle diffusion plot shows two linear portions as represented in Fig. 12D. Film diffusion and external surface adsorption are responsible for the rapid metal ion uptake observed in the initial stage. Followed by slow step, where metal ions are diffused through pores and channels of adsorbent such intra-particle diffusion may evidently control the adsorption process. This rate-limiting step is evidenced by the second straight line in the plot. The third segment represents the equilibrium stage, where site saturation occurs and intra-particle diffusion nears completion^42^. In this case, the intra-particle diffusion remained not the exclusive rate-controlling step, as evidenced by the non-linear plot with a non-zero intercept.

### Adsorption isotherms models

**Fig. 13:**
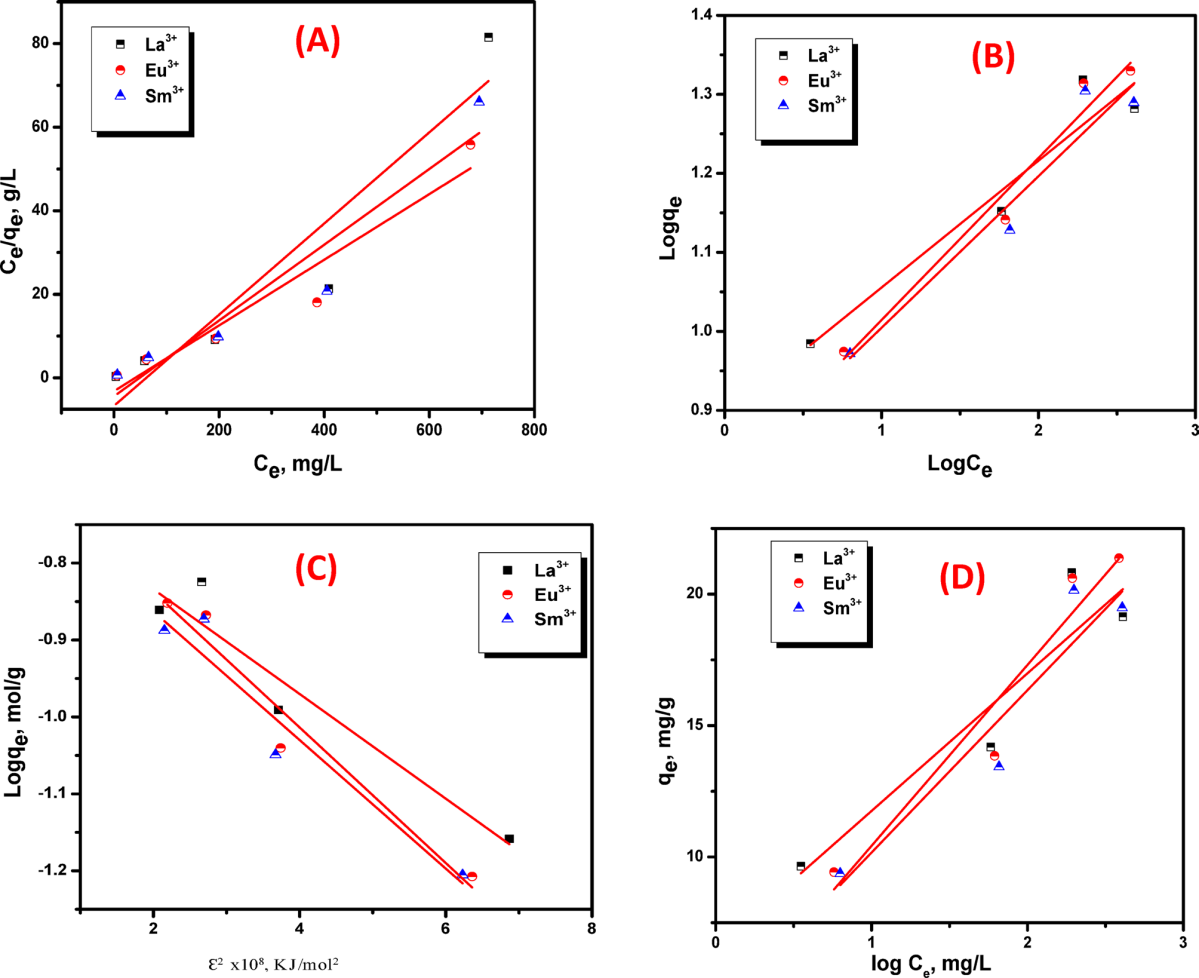
(**A**) Langmuir plots; (**B**) Freundlich plots; (**C**) D-R plots (**D**) Temkin plots for sorption of La^3+^, Eu^3+^ and Sm^3+^ ions onto ZrO_2_.

The study of adsorption isotherms is a fundamental approach for characterizing interfacial reaction mechanisms in solid-liquid systems. An adsorption isotherm refer to the functional relationship, at constant temperature, between *q*_*e*​_ (the adsorption capacity) and *C*_*e*_ (the equilibrium concentration)^43^. In this study, the adsorption data for La³⁺, Eu³⁺, and Sm³⁺ onto ZrO₂ were analyzed using four common isotherm models: Langmuir, Freundlich, Dubinin-Radushkevich (D-R), and Temkin. These models describe the equilibrium distribution of metal ions between the solid adsorbent and the liquid phase. Figure 13A–D shows the linear diagrams for Langmuir, Freundlich, D-R and Temkin isotherms. Each model’s isotherm variables are derived from the relevant graphs and are concise in Table 2. The Langmuir parameters, Q_o_ and b, were gained by linearizing the Langmuir model, with the corresponding plot of C_e_/q_e_ versus C_e_ presented in Fig. 13A. Analysis with the Langmuir model revealed Q_o_ values of 9.16, 12.72, and 11.03 mg/g for the adsorption of La^3+^, Eu^3+^, and Sm^3+^ ions, respectively, onto ZrO_2_. The Langmuir model yielded R² values of approximately 0.85, 0.90, and 0.88 for La^3+^, Eu^3+^, and Sm^3+^ ions, respectively. Evaluation of the isotherms confirmed that the Langmuir model yielded the superior fit to the equilibrium data.

**Table 2:** Langmuir, Freundlich, D-R and Temkin isotherm model parameters for sorption of La(III), Eu(III) and Sm(II) by ZrO_2_.

Isotherm models	Lanthanum	Europium	Samarium
Langmuir parameters			
Q_o_ (mg/g)	9.165	12.72	11.03
b(L/mg) x10^− 3^	16.31	24.78	20.59
R_L_	0.86	0.81	0.97
R^2^	0.859	0.905	0.889
Freundlich parameters			
K_F_(mg^(1−1/n)^L^1/n^g^−1^	7.52	6.44	6.49
1/n	6.22	4.87	5.21
R^2^	0.891	0.952	0.913
D-R parameters			
Q_m_ (mol/g)	4.98	4.57	4.96
K (mol^2^/KJ^2^)x10^− 10^	6.807	8.811	8.343
R^2^	0.888	0.932	0.902
Temkin parameters			
B (KJ/mol)	5.236	6.882	6.178
A (L/g)	17.55	3.271	4.427
R^2^	0.82	0.904	0.855

The dimensionless separation factor (R_L_) offers a more reliable indicator of adsorption and it is used to illustrate the adsorption process favorability. The R_L_ values of La^3+^, Eu^3+^, and Sm^3+^ ions on ZrO_2_ in the current system are 0.86 to 0.81 and 0.97, respectively, suggesting a favorable adsorption process (Table 2).

The Freundlich isotherm is typically used to interpret adsorption processes occurring on heterogeneous surfaces. The value of q_e_ was calculated based on the initial (Co) and equilibrium (C_e_) concentrations, both measured in mg/L. The parameters K_f_ (mg/g) and n were derived from Freundlich plot (log q_e_ vs. log C_e_) in Fig. 13B, utilizing its intercept and slope. The intensity of adsorption is empirically indicated by the Freundlich exponent, which is dimensionless and represented by the symbol n. All n values listed in Table 2 are greater than 1, indicating favorable adsorption conditions for removing La^3+^, Eu^3+^, and Sm^3+^ ions on ZrO_2_ surface.

The adsorption of La^3+^, Eu^3+^, and Sm^3+^ ions on ZrO_2_ can be described by multiple isotherm models, with both Langmuir and Freundlich providing reasonable fits, suggesting a combination of homogeneous and heterogeneous surface interactions.

The adsorption mechanism was further probed by analyzing the data with the Dubinin-Radushkevich (D-R) (Fig. 13C) and Temkin isotherm model (Fig. 13D). The D-R model yielded mean free energies (E) of 12.9, 12.25 and 11.39 kJ/mol for La^3+^, Eu^3+^ and Sm^3+^ ions, respectively (Table 2). Since these exceed 8 kJ/mol, a chemisorption mechanism is indicated^44^. In contrast, the parameters derived from the Temkin isotherm (A_T_ and B, see Eq. 12 and Table 2)) suggest that physisorption also is a contributory factor in the overall process. Where, A_T_ (Temkin equilibrium binding constant) reflects the maximum binding energy of adsorbate–adsorbent interactions, indicating the affinity of the adsorbent surface for the adsorbate and B (Temkin constant related to heat of adsorption) represents the variation of adsorption energy with surface coverage, providing insight into how the adsorption energy decreases linearly as the surface becomes occupied. This explanation clarifies the physical meaning of the Temkin parameters and links them to the adsorption behavior observed in our study.

### Effect of coexisting ions

**Fig. 14:**
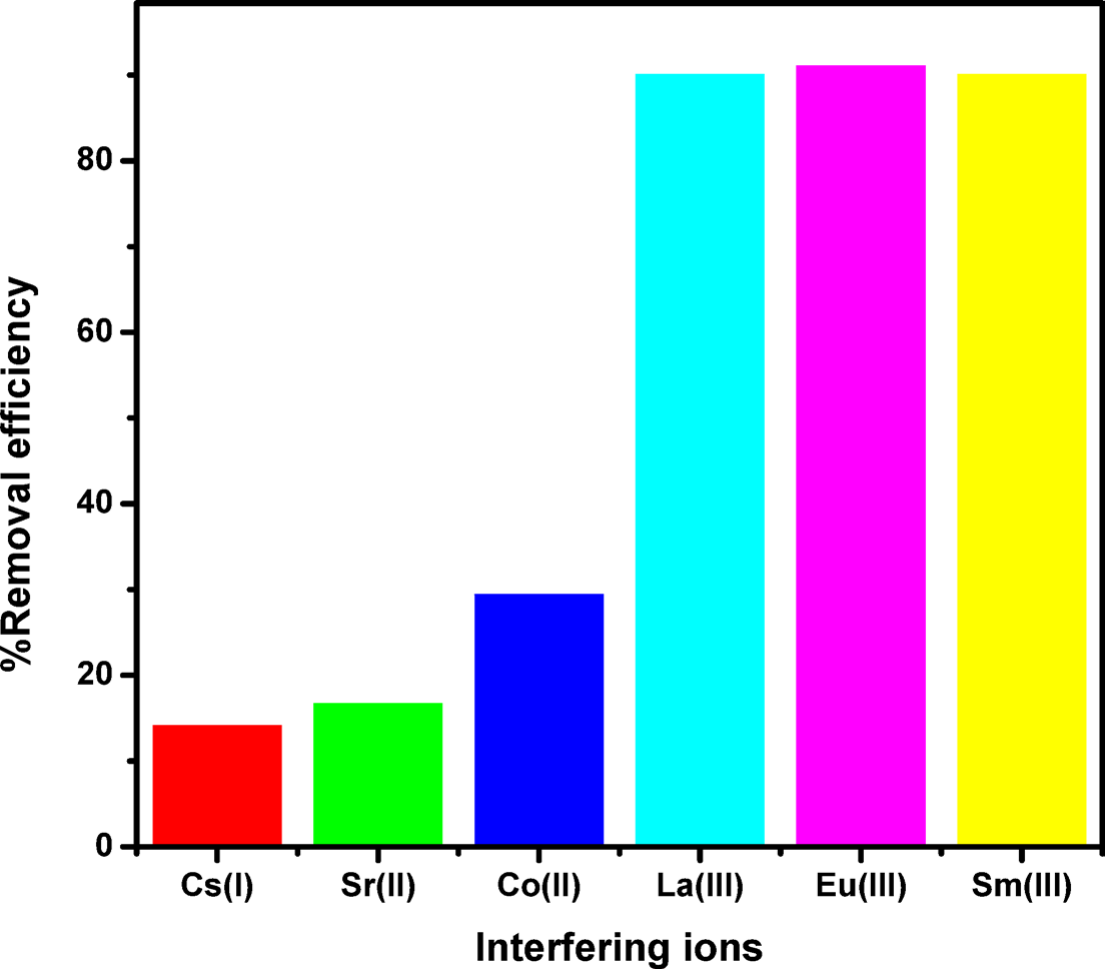
Effect of different coexisting metal ions Cs^+^, Sr^2+^ and Co^2+^ on the adsorption efficiency of La^3+^, Eu^3+^ and Sm^3+^ ions onto ZrO_2_ at initial pH of 3.5.

The consequence of competing ions on the adsorption effectiveness La^3+^, Eu^3+^, and Sm^3+^ ions ions ZrO_2_ can be considered to investigate the capability of ZrO_2_ to remove these target ions in the attendance of other ions. The preferential adsorption of ZrO_2_ for La^3+^, Eu^3+^, and Sm^3+^ ions was evaluated in the presence of competing ions (Cs⁺, Sr²⁺ and Co²⁺). According to the results in (Fig. 14) the adsorption rate of La^3+^, Eu^3+^, and Sm^3+^ ions by ZrO_2_ was (96.48, 94.26 and 93.71%), respectively, which was unaffected significantly by the presence of different ions. The removal effectiveness of the target lanthanides remained high and was not significantly affected by these interferes, demonstrating the adsorbent’s strong affinity.

### Thermodynamic study

**Fig. 15:**
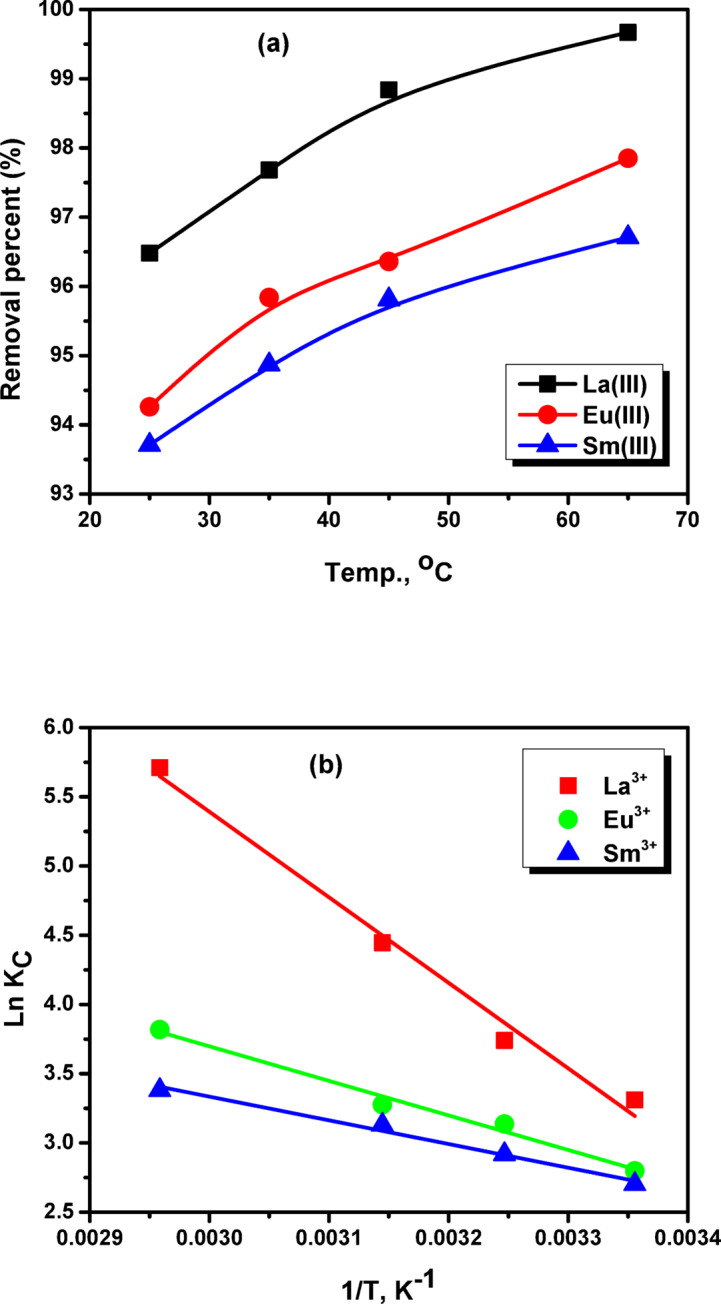
(**a**) the influence of temperature on the removal percentage of La^3+^, Eu^3+^ and Sm^3+^ ions onto ZrO_2_ and (**b**) thermodynamic parameters for adsorption of La^3+^, Eu^3+^ and Sm^3+^ ions onto ZrO_2_ adsorbent.

Temperature is pivotal variable which basically influencing the adsorption operation. The typical energetic profile of adsorption process may be dedicated by determining thermodynamic parameters. The temperature influence of La^3+^, Eu^3+^, and Sm^3+^ ions adsorption onto ZrO_2_ was investigated within the temperature range of 298–338 K. It is evident from Fig. 15a that increasing temperatures significantly increases the removal percent of La^3+^, Eu^3+^, and Sm^3+^ ions onto ZrO_2_. This behavior may discused as raising temperature of aqueous media leads to increasing activation energy of metal ions. However, the possibility of ions diffusion to interacting sites may be increased by increasing temperatures and the removal percent also increased^36^. These results may verify that endothermic processes were responsible for metal ions adsorption onto ZrO_2_. Van’t Hoff’s equations are used to further studying the thermodynamic nature of the sorption operation by calculating standard Gibbs free energy change (ΔG°, kJ/mol), standard enthalpy change (ΔH°, kJ/mol), and standard entropy change (ΔS°, J/(mol·K)). Figure 15b displays La^3+^, Eu^3+^, and Sm^3+^ ions thermodynamic behavior onto ZrO_2_ and detailed constants are recorded in Table 3. The ability and spontaneity of La^3+^, Eu^3+^, and Sm^3+^ ions adsorption onto ZrO_2_ are verified by negative values of ΔG^o^. Additionally, the positive ΔH^o^ values illustrated the endothermic adsorption process. Ultimately, enhanced randomness was devoted by positive values of ΔS^o^.

**Table 3:** Thermodynamic characteristics for adsorption of La(III), Eu(III) and Sm(III) ions onto ZrO_2_.

Metal ions	T, K	ΔG^o^, k J mol^− 1^	ΔH^o^, k Jmol^− 1^	ΔS^o^, J mole^1^K^− 1^
La(III)	298	-18.21	51.63	198.92
	308	-19.57		
	318	-21.73		
	328	-24.84		
	338	-26.91		
Eu(III)	298	-18.01	40.74	92.95
	308	-18.35		
	318	-20.64		
	328	-24.48		
	338	-25.41		
Sm(III)	298	-16.57	34.2	87.33
	308	-17.61		
	318	-20.27		
	328	-22.38		
	338	-23.94		

### Desorption and regeneration studies

**Fig. 16:**
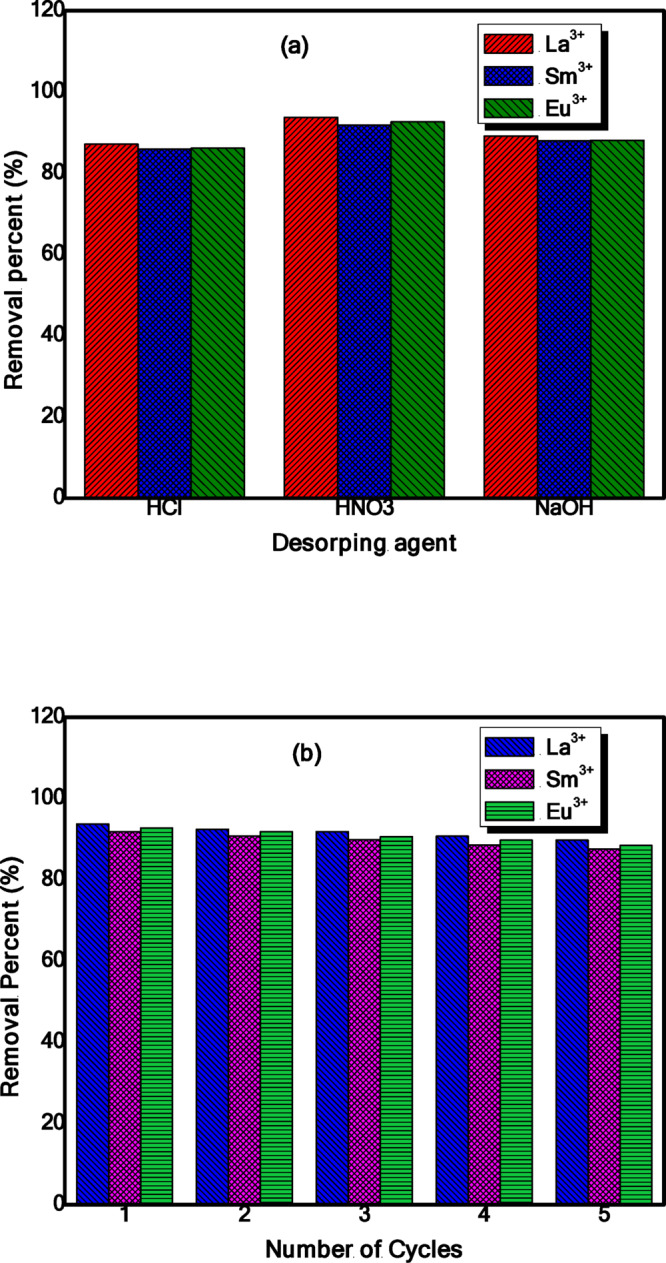
(**a**) desorption efficiency of La^3+^, Eu^3+^ and Sm^3+^ ions using different solutions, (b) recycling efficiency of La^3+^, Eu^3+^ and Sm^3+^ ions onto ZrO_2_ over five cycles.

Desorption studies were conducted to assess the reusability and economic viability of ZrO₂. Herein, La(III)-loaded ZrO_2_, Eu(III)-loaded ZrO_2_ and Sm(III)-loaded ZrO_2_ were preserved with 0.1 M solutions of [HCl, HNO_3_, and NaOH] at 25 °C for 4 h. Analysis of the resulting solutions revealed that La^3+^, Eu^3+^, and Sm^3+^ recovery efficiencies of 93.75, 91.85%, and 92.65%, respectively, with 0.1 M HNO₃ proving to be the most effective eluent (Fig. 16a). Given the industrial importance of lanthanide ions (Ln(III)), their recovery from waste streams is a critical factor for practical application. To assess reusability, five consecutive adsorption-desorption cycles were performed. In each cycle, ZrO_2_ was loaded with La^3+^, Eu^3+^, and Sm^3+^ ions under standard adsorption conditions, separated via filtration, and then eluted with 0.1 M HNO₃ based on the optimal desorption results. The regenerated ZrO₂ was rinsed with water before being reused in the subsequent cycle.

The regeneration efficiency over these five cycles is displayed in Fig. 16b. The adsorption capability for La^3+^, Eu^3+^, and Sm^3+^ ions remained stable, with only a slight decrease observed after the fourth cycle. This minimal loss in performance suggests that the 0.1 M HNO₃ eluent effectively recovers La^3+^, Eu^3+^, and Sm^3+^ ions and regenerates ZrO_2_ without significantly degrading its structure^46^. The minor decline in capacity is likely attributable to a gradual weakening of electrostatic interactions after prolonged cycling, rather than a loss of adsorbent integrity. The extraordinary desorption efficacy and sustained performance over numerous rounds demonstrate that ZrO_2_ possesses outstanding regeneration capacity, underscoring its potential as a cost-effective sorbent for practical lanthanide recovery applications^45^.

### Comparison with other studies

The greatest adsorption capacity of ZrO_2_ for La^3+^, Eu^3+^, and Sm^3+^ ions in comparison to other adsorbents is shown in Table 4. ZrO_2_ has a significant ability to adsorb La^3+^, Eu^3+^, and Sm^3+^ ions in comparison to the literature^45–63^.

**Table 4:** Comparison between the adsorption capacity of La (III), Eu(III) and Sm(III) ins onto ZrO_2_ with various adsorbents.

Adsorbent	Condition			q (mg/g)			Reference
	pH	Temp. ^o^C	C_o_, mg/L	La(III)	Eu(III)	Sm(III)	
Dowex 50 W-X8	5.5	25	60	3.6	-	-	^45^
IIP-AM	3	25	1050	9.07	-	-	^46^
activated carbon (AC)	4.5	Room temp.	1083	11.4	-	-	^47^
Magnetic nanoparticles functionalized with Diethylenetriamine pentaacetic acid (DTPA)	3	-	-	0.091	-	1.35	^48^
Chitosan-functionalized magnetite-pectin	5	25	25	8.17	-	-	^49^
ZrO_2 _	3.5	25	100	9.16	12.72	11.03	this study
Organomodified red clay	6	25	-	19.46	-	-	^50^
Cysteine – chitosan magnetic nanoparticle	5	25	-	16.0	-	-	^51^
Fe-modified biochar	5	22	-	-	9.8	-	^52^
Bentonite modified with N-(2-hydroxyethyl)ethylenediamine	4	-	-	-	-	17.7	^53^
Hydroxyapatite	5.7	20	9.87	0.25	0.94	-	^54^
Silica-based urea–formaldehyde impregnated with organophosphorus extractant	3	25	151	-	3.1	-	^55^
SiO_2_	3	25	151	-	0.12	-	^55^
Silica-based urea–formaldehyde composite (SiO_2_/UF )	3	25	151	-	0.23	-	^55^
TiO_2_- fulvic acid	4.5	20	0.152	-	0.017 m mol/g	-	^56^
SBA-15 mesoporous silicas functionalized with N-propyl salicylaldimine	4	25	10	-	5.1	-	^57^
Crab shells	4	25	100	-	3.23	-	^58^
Raw cactus fiberes _2_	4	23	1.51	-	0.16	-	^59^
Modified cactus fibres (Phosphorylated)	4	23	1.51	-	0.045	-	^59^
Modified cactus fibres (MnO_2_-coated))	4	23	1.51	-	0.46	-	^59^
Amberlite XAD-7 resin impregnated with DEHPA extractant	6	25	200	5.52	-	-	^60^
GA-g-PAM/SiO_2_	-	-	-	7.9	-	-	^61^
Zirconium triethylenetetramine (ZrT)	4–5	-	-	6.07	-	-	^62^
Amberlite IRC-50 (H^+^)	9.3	20	1 mmol/L	-	-	22.2	^63^

Although the adsorption capacities of the ZrO_2_ adsorbent for La³⁺, Eu³⁺, and Sm³⁺ are moderate relative to some previously reported high-capacity adsorbents, it is important to consider differences in experimental conditions, pH, initial concentrations, and adsorbent preparation. For instance, some high-capacity adsorbents require complex synthesis routes or harsh chemical treatments, which may limit their practical applicability. The present zirconium oxide adsorbent offers several practical benefits. It was prepared from commercially available zirconium precursors using a simple synthesis approach. The synthesis is environmentally benign, avoiding toxic reagents or high-energy processing. Moreover, the adsorbent maintains high adsorption efficiency over multiple adsorption–desorption cycles, demonstrating operational stability. The material can be applied directly in aqueous systems without extensive pretreatment or modification. Overall, the adsorption capacities are moderate, the combination of adequate performance, simplicity, sustainability, and reusability of zirconium oxide as a practical and attractive adsorbent for La³⁺, Eu³⁺, and Sm³⁺ recovery from acidic media.

## Conclusion

In this study, zirconium oxide (ZrO_2_) was fruitfully produced via an environmentally friendly synthesis route and comprehensively characterized by SEM-EDX, XRD, TGA/DTA, and FT-IR techniques. The prepared adsorbent was effectively employed for adsorption of trivalent lanthanides, La^3+^, Eu^3+^, and Sm^3+^ ions from acidic solution. Investigations into the effect of pH revealed it to be a highly influential factor on adsorption efficacy. Notably, a consistent optimum pH of 3.5 was determined for the uptake of La^3+^, Eu^3+^, and Sm^3+^ ions onto ZrO_2_. Kinetic data exposed that equilibrium was attained within 60 min, and the process was excellent conformity to the pseudo-second-order model, implying that chemisorption was the primary rate-controlling step. Furthermore, equilibrium isotherm data were most accurately described by multiple isotherm models, with both Langmuir and Freundlich providing reasonable fits, suggesting a combination of homogeneous and heterogeneous surface interactions The thermodynamic parameters indicated that the sorption process is both endothermic and spontaneous. Moreover, La^3+^, Eu^3+^, and Sm^3+^ ions can be readily desorbed from the loaded samples using 0.1 M HNO_3_, with efficient recyclability maintained over five successive cycles. In summary, the green-synthesized ZrO_2_ demonstrates significant potential as an effective and supportable adsorbent for rare earth elements recovery from acidic streams.

## Data Availability

All data generated or analyzed during this study are included on this article.
